# Activation of hepatic stellate cell in *Pten* null liver injury model

**DOI:** 10.1186/s13069-016-0045-1

**Published:** 2016-06-14

**Authors:** Lina He, James Gubbins, Zhechu Peng, Vivian Medina, Fan Fei, Kinji Asahina, Jiaohong Wang, Michael Kahn, Carl B. Rountree, Bangyan L. Stiles

**Affiliations:** Pharmacology and Pharmaceutical Sciences, School of Pharmacy, University of Southern California, Los Angeles, CA 90033 USA; Imperial College, London, England; Pathology, Keck School of Medicine, University of Southern California, Los Angeles, CA 90033 USA; Department of Biochemistry and Molecular Biology, Keck School of Medicine, University of Southern California, Los Angeles, CA 90033 USA; Department of Pediatrics and Pharmacology, Pennsylvania State University College of Medicine, 500 University Drive, H085, Hershey, PA 17033 USA; Pharmacology and Pharmaceutical Sciences, USC School of Pharmacy PSC402, 1985 Zonal Ave, Los Angeles, CA 90089 USA

**Keywords:** Fibrosis, Wnt, PTEN, AKT, Fatty liver, Steatosis

## Abstract

**Background:**

Hepatic fibrosis is a prominent pathological feature associated with chronic liver disease including non-alcoholic hepatosteatosis (NASH), and a precursor for liver cancer development. We previously reported that PTEN loss in the liver, which leads to hyperactivated liver insulin signaling results in NASH development. Here we used the same mouse model to study the progression from steatosis to fibrosis.

**Results:**

The *Pten* null livers develop progressive liver fibrosis as indicated by Sirius Red staining and increased expression of collagen I, Timp 1, SMAα, and p75NTR. Consistently, hepatic stellate cells (HSCs) isolated from *Pten* null livers are readily activated when compared with that from mice with intact PTEN. Deletion of AKT2, the downstream target of PTEN signal, blocked NASH development, and alleviated fibrosis. HSCs from the *Pten/Akt2* double null mice are quiescent like those isolated from the control livers. Our analysis shows that the activation of HSCs does not depend on the intrinsic signals regulated by PI3K/AKT, the target of PTEN, but does depend on steatosis and injury to the liver. During the progression of liver fibrosis in the *Pten* null model, Wnt ligands and signaling receptor are induced, concurrent with the reduction of sFRP5, a Wnt antagonist. We showed that treatment of HSCs with Wnt receptor antagonist blocks the observed morphological changes when HSCs undergo activation in culture. This signal appears to be mediated by β-catenin, as manipulating β-catenin signaling alters marker gene expressions of HSC activation.

**Conclusions:**

Wnt/β-catenin activation serves as an important mediator for fibrosis development resulting from NASH using a mouse model where NASH is mimicked by PTEN loss.

## Background

Fatty liver disease (FLD) is the most prevalent form of chronic liver disease and has become a major pandemic in developed and some developing countries. FLD is characterized by lipid accumulation in the liver resulting from sedentary lifestyle, calorie-rich diet, or alcohol consumption. Without intervention, FLD can develop into non-alcoholic or alcoholic hepatosteatosis (NASH or ASH) where fatty liver is accompanied by infiltration of inflammatory cells. Left untreated, NASH and ASH can develop into fibrosis, a common late stage chronic liver disease and a significant risk factor for cancer [1].

The mechanisms responsible for this fibrosis development in NASH/ASH patients are not well studied but may involve injury-repair responses and accumulation of myofibroblasts. Myofibroblasts are thought to be the cells that lay down the collagens that compose the fibrotic tissue [2]. Of the potential sources for these myofibroblasts, activation of hepatic stellate cells (HSCs) has gained strong support [3]. Inactive HSCs are the main storage for vitamin A. Upon injury, HSCs *activate* by losing their vitamin A containing lipid droplets and gaining myofibroblast characteristics. This morphological alteration is associated with increased expressions of markers such as, collagen type I (Col1a1), smooth muscle actin α (SMAα), desmin, and nerve growth factor receptor (P75NTR). Multiple stimuli and pathways have been shown to stimulate the expression of these markers in HSCs. The most prominent among these are transforming growth factor β (TGF β) and platelet-derived growth factor (PDGF) [4].

To investigate the molecular signals involved in fibrosis resulting from fatty liver, we used a murine model where steatosis is induced as a result of hyperactivated insulin signal, a condition that commonly occurs in NASH patients. In this mouse model (hereafter referred to as *Pten* null mice), *Pten* (phosphatase and tensin homologue deleted on chromosome 10) is deleted in the albumin-positive cell population (*Pten*^*loxP/loxP*^; *Alb-Cre*^*+*^*)*. In such mice, the liver PI3K/AKT pathway responsible for transmitting insulin signal is induced without the complication of peripheral insulin resistance and hyperglycemia [5]. The *Pten* null mouse model thus mimics the liver signals occurring in NASH patients where hyperinsulinemia often drives lipid synthesis in the liver. In this model, we have previously observed significant injury to the liver and ultimately liver tumor development [5–7]. In the current study, we characterize the fibrosis development in *Pten* null mice and validated it as an appropriate model to study the contribution of NASH to fibrosis. In addition, we investigated the mechanisms underlying the steatosis contribution to fibrosis development using this model.

## Results

### PTEN levels are lower in NASH patients

**Fig. 1:**
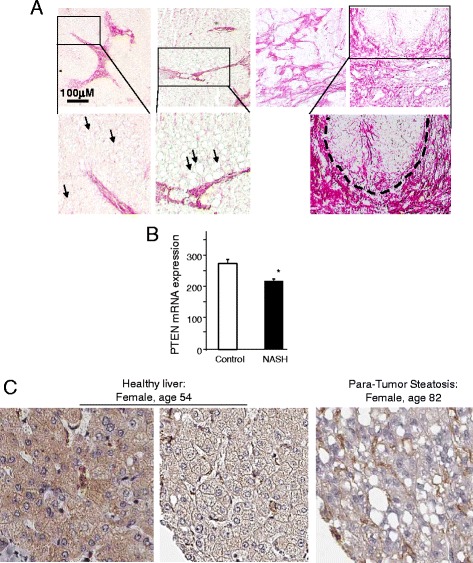
Association of fibrosis and PTEN status with NASH. **a** Sirius Red staining for fibrosis in biopsied liver human non-alcoholic steatohepatitis (NASH) patients. Biopsy livers were obtained from patients with various degrees of diagnosed NASH. Sirius Red staining is performed on the livers to identify fibrotic regions. *Top two panels*, fibrosis near fatty liver deposits; *bottom up second panel*, premalignant lesions with intensive Sirius Red staining; *bottom panel*, areas with fibrotic tissues only. *Right panels*, higher magnified images of the cropped areas from the *left panels. Arrows*: lipid deposit in hepatocytes. *Dotted enclosure*, premalignant lesion. **b** PTEN expression is lower in NASH vs. control samples. Publically available data set (GSE37031) was analyzed for expression of PTEN. *p < 0.05. **c** Immunohistochemical analysis of PTEN protein in two patients. Images generated by The Human Protein Atlas (www.proteinatlas.org)

Liver fibrosis/cirrhosis often accompanies the development of fatty liver as confirmed here with Sirius Red staining (Fig. 1a). Using a published data set (GSE37031) [8], we found that expression of PTEN, a lipid phosphatase is negatively correlated with the presence of NASH (Fig. 1b). The protein expression of PTEN is further verified by analyzing images obtained from the Human Protein Atlas (http://www.proteinatlas.org). Within healthy liver, PTEN expression is lower in hepatocytes with micro-vesicular lipid droplets (Fig 1, left two panels). In steatotic liver tissues from HCC patient, PTEN expression is only detected in non-hepatocytes (Fig. 1c).

### *Pten* null mice develop progressive liver fibrosis

**Fig. 2:**
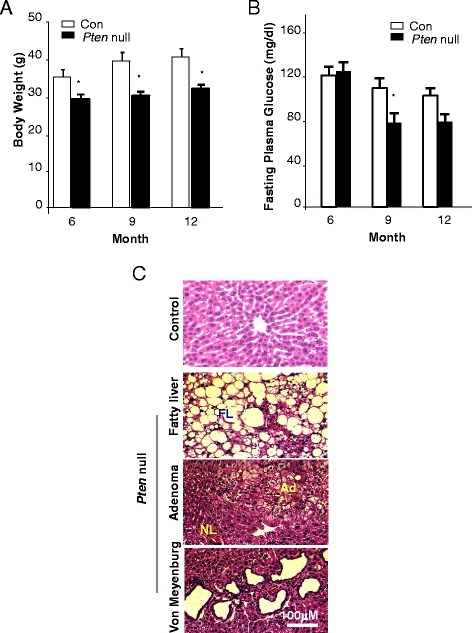
Phenotypes of mice carrying the deletion of *Pten* (*Pten* null) in the liver. **a** Body weight of control and *Pten* null mice. **b** Fasting plasma glucose in control and *Pten* null mice. *n* = 7–10. *p < 0.05. **c** Liver-specific *Pten* deletion mice develop fatty liver disease at an early age (*top*; *FL* fatty liver). Adenomas are also observed in these mice (*middle*, *nl* normal liver, *Ad* adenomas). Morphologies that resemble Von Meyenburg syndrome are also observed in the livers of the liver *Pten* null mice (*bottom*)

**Fig. 3:**
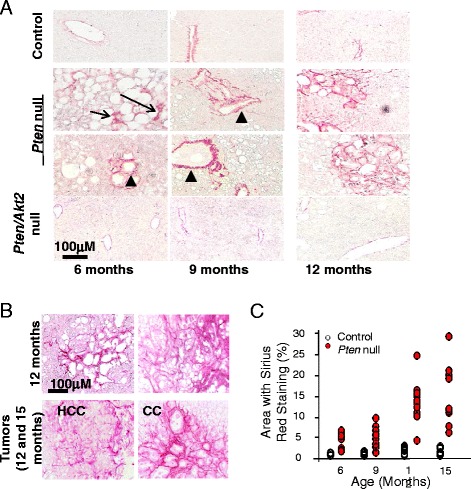
*Pten* null livers develop progressive fibrosis that is attenuated by AKT2 loss. **a** Periductal (*arrowhead*) and pericellular (*arrows*) staining of Sirius Red are obvious in the 6- and 9-month-old *Pten* null livers, indicating fibrosis development. In older mice (12 months of age), more intensive Sirius Red staining is observed. In *Pten* and *Akt2* double mutants (*Pten/Akt2* null), minimum staining for Sirius Red is observed (*bottom row*). *Top row*, controls; *middle two rows*, *Pten* null livers. **b** Sirius Red staining in tumor samples from the *Pten* null mice. **c** Sirius Red stained areas were quantified vs. non-stained areas. Percentage of total area that is positive for Sirius Red is reported. *Each circle* represents an animal. *Open circle*, control; *solid red circle*, *Pten* null

In mouse models, PTEN loss in the liver leads to hyperactivation of the PI3K/AKT pathway, leading to progressive NASH followed by spontaneous tumor development [5–7]. Consistent with our previous results in young mice [5], the older *Pten* null mice in the current study displayed lower body weight and plasma glucose (Fig. 2a, b) throughout all age cohorts. At 6 months of age, all mice exhibit fatty liver disease with adenomas and hyperproliferation of the ductal epithelial similar to Von Meyenburg syndrome (Fig. 2c). Pericellular staining of Sirius Red is observed in areas of steatosis at this age (Fig. 3a) and becomes progressively more severe in 9- and 12-month-old *Pten* null mice (Fig. 3b, c), consistent with clinical observations where fibrosis accompanies steatosis. In addition to *Pten* null mice, we also evaluated Sirius Red in a model in which both *Pten* and *Akt2* are deleted. The deletion of *Akt2* eliminates the occurrence of NASH that develops in mice lacking PTEN alone [7]. Consistent with the lack of NASH status of the *Pten*/*Akt2* double-deleted mice, only isolated ducts are stained with Sirius Red in the *Pten/Akt2* double null mice, similar to the control livers (Fig. 3a). Together, our data suggests that PTEN loss leads to fibrosis development and AKT2 plays a role in this development.

**Fig. 4:**
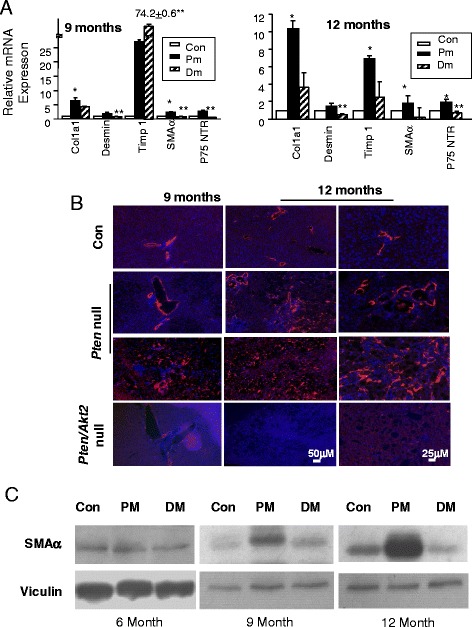
Fibrosis markers are upregulated in the *Pten* null livers and reduced when *Akt2* is simultaneously deleted. **a** Expression analysis of markers for fibrosis, i.e., desmin, SMAα, Timp 1, Col1a1, and p75NTR are analyzed in 9 (*left*)- and 12 (*right*)-month-old *Pten* control (*Con*) and null livers (*Pm*) as well as the *Pten/Akt2* double null livers (*Dm*). *Solid bars*, *Pten* null liver; *stripped bars*, *Pten/Akt2* double null; *open bars*, control liver. *Striped bars*, *Pten/Akt2* double null. *significantly different from controls of the same gene analyzed; **significantly different from *Pten* null group of the same gene analyzed. *n* = 5. *p* < 0.05. Three animals each group are used for the experiment with each sample repeated twice. **b** Liver tissues were stained with SMAα antibody using indirect immunofluorescence staining (*red*). The same slides were counterstained with DAPI (*blue*) to visualize nucleus. *Left panels*, 9-month-old livers; *middle panels*, 12-month-old livers; *right panels*, high mag images of 12-month-old livers. *Top panels*, control; *middle two rows*, *Pten* null; *bottom row*, *Pten* and *Akt2* double mutant (*Pten/Akt2* null). **c** Immunoblotting analysis of SMAα in liver lysate isolated from the indicated mouse models. *Pten* control (*Con*) and null livers (*Pm*) as well as the *Pten/Akt2* double null livers (*Dm*)

To further confirm the fibrosis pathology, we analyzed the expression of several fibrosis markers: Col1a1, desmin, SMAα, and p75NTR as well as tissue inhibitor of metalloproteases 1 (Timp 1) to quantitatively assess the buildup of collagen fibers. We show that expressions of these markers are significantly upregulated in 9- and 12-month-old *Pten* null livers (Fig. 4a). Consistent with a role of AKT2 in the development of fibrosis, the expression of these fibrogenic genes were reduced when *Akt2* is deleted simultaneously with *Pten*. The only exception is Timp 1 expression in 9-month-old mice. The expression of Timp 1 increased by two- to threefolds in *Akt2*/*Pten* double mutants vs. *Pten* deletion alone. The lack of downregulation in Timp 1 by *Akt2* deletion and its decline in 12- vs. 9-month-old mice may be related to its potential role in tumorigenesis as all *Pten* null mice developed tumors at 12 months of age and Timp 1 has been shown to inhibit mitogenesis in HCC [9].

To confirm these gene expression changes, we stained liver sections for SMAα. In 9-month-old *Pten* null mice, this staining is mostly observed in areas of severe fatty liver and the ductal plates (Fig. 4b). In 12-month-old mice, SMAα staining is distributed throughout the *Pten* null livers, whereas the *Pten/Akt2* double null and control livers have little staining for SMAα at either age. The SMAα staining is further confirmed with immunoblotting analysis showing increased SMAα in 9- and 12-month-old mice (Fig. 4c). These observations show that *Pten* deletion leads to progressive and severe fibrosis that is recovered when AKT2 is simultaneously lost.

### HSCs are activated in *Pten* null liver

**Fig. 5:**
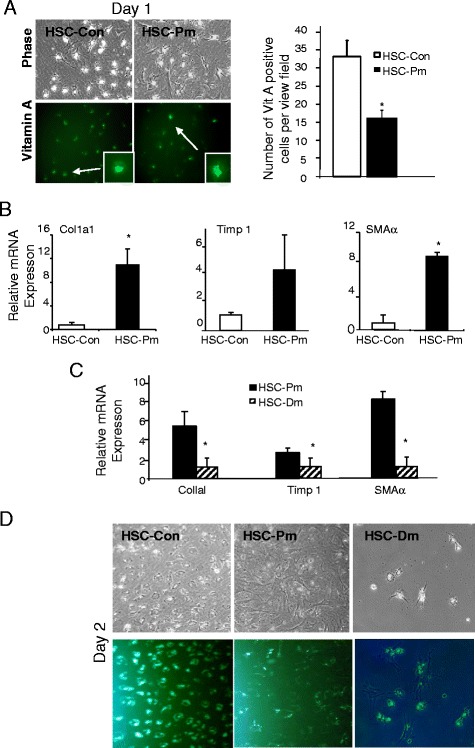
Hepatic stellate cells (HSCs) from the *Pten* null mice are activated. **a** Morphology of HSCs isolated from *Pten* control (HSC-Con) and null (HSC-Pm) mice after overnight culturing for attachment. *Top left panel*, phase contrast images; *bottom left panels*, autofluorescence of vitamin A. Green fluorescence (*pseudo-colored green*) indicates vitamin A-rich oil droplets. *Inset*, higher magnification images of the oil droplets with vitamin A autofluorescence. *Right panel*, quantification of vitamin A-positive cells. *significantly different between the two groups at *p* < 0.05. At least three randomly selected views per culture were used for measurement. **b** Expression of fibrosis markers (Col1a1, SMAα, and Timp 1) indicates that HSCs from *Pten* null livers are activated. *Open bar*, HSCs isolated from control livers (HSC-Con); *solid bar*, HSCs isolated from *Pten* null livers (HSC-Pm). *significantly different between the two groups at *p* < 0.05. *n* = 3. **c** Expression of fibrosis markers in HSC isolated from the *Pten* null (HSC-Pm) and *Pten/Akt2* double null (HSC-Dm) livers. *significantly different between the two groups at *p* < 0.05. *n* = 3. **d** HSCs from *Pten* null livers are fully activated at day 2 of culture where HSCs from control (HSC-Con) and *Pten/Akt2* double null (HSC-Dm) livers still remain quiescent. *Top panel*, phase contrast images; *bottom panel*, autofluorescence of vitamin A (*pseudo-colored green*)

The buildup of collagen fibers and upregulation of Col1a1 and Timp 1 have been attributed to the activation of HSCs. We isolated HSCs from the 9-month-old *Pten* null (HSC-Pm) and control livers (HSC-Con). The majority of the HSC-Con cells displayed rounded shapes and intense vitamin A autofluorescence, indicating quiescent HSCs (Fig. 5a). HSC-Pm was a mixture of rounded quiescent HSCs and cells with elongated myofibroblast morphologies (Fig. 5a). Expressional analysis of markers indicates that Col1a1 (5.4 × 10^−2^ vs. 5.9 × 10^−3^) and SMAα (1.9 × 10^−3^ vs. 2.2 × 10^−4^) are more than fivefold higher in HSC-*Pten* null cells when compared with HSC-Con (Fig. 5b). Timp 1 expression is also threefold higher in HSC-Pm vs. HSC-Con. Expression of these markers is significantly reduced in HSC isolated from the *Pten/Akt2* double null mice (HSC-Dm) compared to their expression in HSC-Pm cells (Fig. 5c). No difference in p75NTR and desmin expression was observed in the freshly isolated HSCs (data not shown).

The morphological differences between the two HSC cultures are more obvious after the attachment of HSCs at day 2 of culturing (Fig. 5d). The majority of the HSC-Con cells are still round-shaped quiescent HSCs retaining vitamin A autofluorescence, whereas most HSC-Pm cells acquired spindle morphology with very little vitamin A deposits, indicating that they are activated HSCs. HSC-Dm displayed similar quiescent phenotype and retained vitamin A autoflorescence. Like reported, HSC-Con gradually changed from the round-shaped quiescent HSC phenotype to the long, stretched, spindle phenotype that resembles activated HSC from days 3 to 7 (data not shown). By day 5, majority of the HSC-Con are activated and by day 7, all cells display the elongated spindle morphology. HSCs from *Pten* null mice changed very little morphologically as they already acquired the spindle morphology by day 2. The cells appear to shrink with time, likely due to increasing cell density with culturing (data not shown). Consistent with the morphological changes occurring in HSC-Con culture, expressional analysis of Col1a1, Timp 1, and SMAα increased with culturing (data not shown).

### Activation of HSC in *Pten* null mice depends on injury but not intrinsic PI3K/AKT activation

**Fig. 6:**
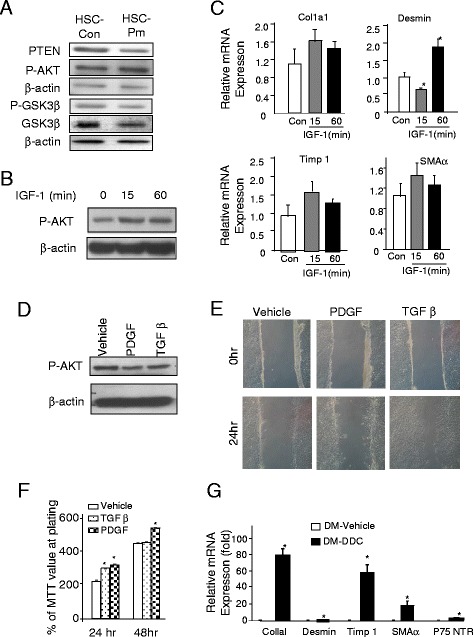
PTEN regulates HSC gene expression independent of AKT signal. **a**. Protein analysis of HSCs isolated from control (HSC-Con) and *Pten* null (HSC-*Pten* null) livers. Membrane is probed with PTEN, p-AKT, p-GSK3β, GSK3β, and α-actin for loading control. **b** Induction of AKT phosphorylation by IGF-1 treatment in human HSC cell line. β-actin is used as loading control. **c** Quantitative PCR analysis of expression of SMAα, Col1aI, Timp 1, and desmin in vehicle- and IGF-1 (*gray bar*, 15 min; and *black bar*, 1 h)-treated samples, respectively. *n* = 3, *p < 0.05 when compared to the vehicle-treated group. **d** Analysis of AKT phosphorylation in response to PDGF (50 ng/ml) and TGFβ (4 ng/ml) treatment. **e** PDGF and TGFβ treatment induced HSC migration as measured with a wound healing assay. Representative images of three experiments. **f** PDGF and TGFβ both induced cell proliferation in cultured HSCs. Cell proliferation is measured by a MTT assay. *n* = 3, *p < 0.05. **g** Relative expression of SMAα, Col1aI, Timp 1, p75NTR, and desmin in livers of *Pten* and *Akt2* double mutant (Dm) mice treated with (*black bar*) or without DDC (*open bar*). *n* = 5, *p < 0.05 when compared to the vehicle-treated group

Constitutively activation of AKT results in induction of collagen I [10] whereas the introduction of dominant negative PI3K inhibits Timp 1 and SMAα [11]. We evaluated whether PI3K/AKT signal may be chronically activated in HSC-Pm isolated from the *Pten* null mice and could be responsible for the phenotypes. While the levels of PTEN decreased moderately in the HSC-Pm, the levels of p-AKT did not differ significantly between HSC-Pm and HSC-Con cells (Fig. 6a). Furthermore, levels of p-GSK3β, a substrate of AKT did not increase either. Instead, p-GSK3β and GSK3β levels are slightly lower in the HSCs from *Pten* null mice. This observation suggests that intrinsic signals due to PI3K activation are unlikely to play a role in the activated HSC observed in the *Pten* null liver. Thus, HSC activation phenotype in *Pten* null mice is likely independent of the intrinsic PI3K signaling pathways.

To further confirm this observation, we treated rat HSC cell line with insulin growth factor (IGF-1) to induce PI3K/AKT. In these cells, overnight starvation led to moderate downregulation of p-AKT. When IGF-1 is added, AKT phosphorylation is induced both 15 min and 1 h after the start of the treatment (Fig. 6b). Phosphorylation of AKT starts to recover after 1 h (data not shown). We evaluated the expression levels of SMAα, Col1a1, Timp 1, and desmin (Fig. 6c). The expression of most fibrotic genes did not alter significantly when p-AKT is induced in the IGF-1 treated HSCs with the exception of desmin expression. At 15 min, we observed a significant downregulation of desmin whereas this level increased after 1 h of treatment. On the other hand, treatment with PDGF and TGFβ both induced migration of the cultured HSCs but did not show significant increase on p-AKT (Fig. 6d–f). These data, together with the moderate change observed for PTEN and p-AKT expression in HSCs isolated from *Pten* null livers (Fig. 3a), indicate that the intrinsic signal due to PTEN loss in HSCs is an unlikely mechanism for their activation in the injured *Pten* null liver and that fibrosis is likely due to secondary effects, e.g., resulting from the underlying liver injury.

To test this in vivo, we induced injury in the *Pten*/*Akt2* double null mice where injury does not occur spontaneously. Inducing injury with DDC in these mice led to robust expression of markers for fibrosis (Fig. 6g), including Col1a1, Timp 1, and SMAα. Expression of desmin and p75 NTR is also moderately induced by treatment of DDC. This observation suggests that the induction of these genes do not rely on the signals of AKT2 but do require injury.

### Wnt signaling mediates HSC activation in injured steatotic *Pten *null liver

**Fig. 7:**
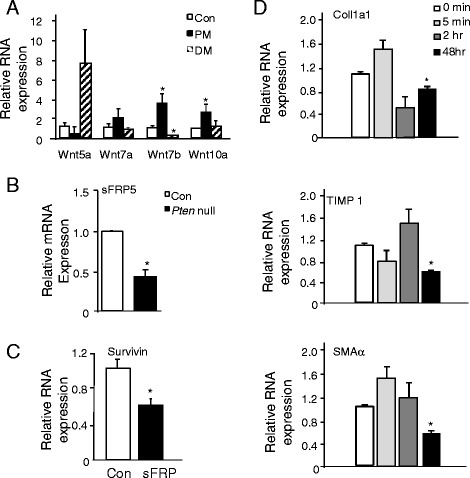
Activation of β-catenin leads to the activation of HSCs. **a**. Expression of Wnt ligands Wnt5a, 7a, 7b, and 10a in control (*Con*), *Pten* null (*Pm*), and *Pten/Akt2* double mutant (*Dm*) livers. *significantly different from the control groups of the same gene at *p* < 0.05. *n* = 3. **b** Expression of Wnt 5a alternative receptor sFRP5 in the control (*Con*, *open bar*) and *Pten* null liver (*solid bar*). *significantly different from the control groups of the same gene at *p* < 0.05. *n* = 5. Cultured human HSCs were treated with sFRP, and expression of fibrogenic genes was evaluated (**c**, **d**). **c** Survivin expression indicating that sFRP treatment reduced β-catenin transcriptional activity. **d** Expression of fibrogenic genes. *significantly different from the control groups of the same gene at *p* < 0.05. *n* = 3

We showed previously that AKT2 loss inhibits liver injury induced by *Pten* deletion [6]. In the same study, we also showed that expression of Wnt7a and 10a and Fzd2, a Wnt receptor, is induced in the *Pten* null liver [6]. Wnt signal may be a potential activator of fibrosis [12, 13]. To test the hypothesis that lipotoxic injury induces Wnt to promote fibrosis development, we first confirm that with the exception of Wnt 5a, Wnt 7a, 7b, and 10a expressions are all induced in 9-month-old *Pten* null steatotic livers and returned to control levels or lower in the non-steatotic *Pten*/*Akt2* double null livers where *Pten* is still deleted and *Akt2* loss rescued steatosis (Fig. 7a). We also found that though Wnt 5a levels did not increase, sFRP5a levels are significantly reduced (Fig. 7b).

**Fig. 8:**
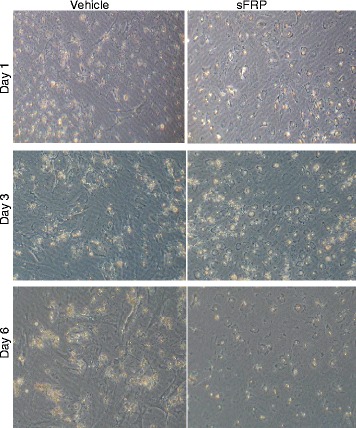
Images of cultured HSCs treated with or without sFRP. HSCs isolated from control mice are treated with sFRP on the third day of culture for 1 day (day 1), 3 days (day 3), and 6 days (day 6). sFRP treatment completely blocked the activation of HSCs at D3 and D6. The morphology of HSCs in sFRP-treated cultures did not progress to spindle fiber whereas obvious spindle fiber formation is observed in the vehicle-treated cultures

Wnt 5a was found to inhibit the differentiation and accumulation of lipid in adipocyte and favors the elongated morphology, whereas its inhibitor soluble fizzled-related protein 5a (sFRP5a) does the opposite [14–16]. To test whether aWnt5a-sFRP5a-like signaling may play a role in the activation of HSCs, we treated cultured rat HSCs with a human recombinant sFRP and investigated the expressions of Col1a1, SMAα, and Timp 1 (Fig. 7c, d). While the time course response is different for each marker gene, the expression of Col1a1, SMAα, and Timp 1 all went down at 48-h posttreatment. Expression of desmin and p75NTR did not differ within this time frame (data not shown). A longer time window may be needed to observe changes in the latter two markers. In addition, treatment of HSC-Con culture with sFRP blocked the morphological switch of HSCs (Fig. 8). The HSC-Con culture remained round quiescent 6 days after the addition of sFRP, whereas the spindle-shaped activated HSC morphology progressed in the vehicle-treated cells. In the HSC-Pm culture, treatment with sFRP was ineffective (data not shown), likely because the HSCs are already activated. This data suggests that downregulation of sFRP5a and upregulation of Wnt ligands observed in the *Pten* null liver may have permitted the activation of HSCs in vivo.

**Fig. 9:**
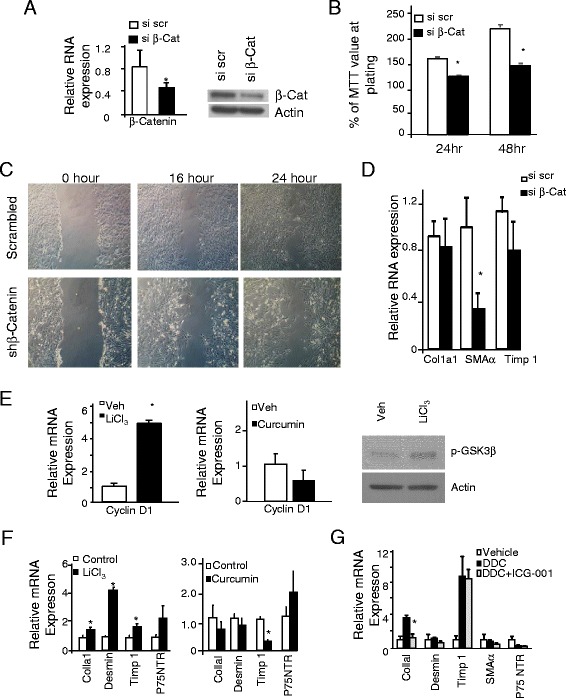
Inhibition of Wnt signal blocks the activation of HSCs. **a** Downregulation of β-catenin with siRNA against β-catenin led to reduced expression of fibrogenic genes. *Left*, β-catenin mRNA expression in si-scrambled and si-β-catenin transfected HSCs. *Right*, protein levels of β-catenin in si-scrambled and si-β-catenin transfected HSCs. *significantly different from the control groups of the same gene at *p* < 0.05. *n* = 3 with experiments repeated three times. **b** The effects of β-catenin knockdown on cell proliferation measured by a MTT assay. *n* = 3, *p < 0.05. **c** Representative images of wound healing assay in shβ-catenin knockdown cells vs. controls. **d** mRNA expression of marker genes of fibrosis in si-scrambled and si-β-catenin transfected HSCs. **e** LiCl_3_, an activator of β-catenin, and curcumin, an inhibitor of β-catenin activity, are used to treat primary HSCs at day 2 of culture. *Left panel*, LiCl_3_ treatment led to the activation of β-catenin transcriptional activity indicated by an induction of cyclin D1 expression. *Middle panel*, curcumin treatment led to attenuated cyclin D1 expression, indicating the inhibition of β-catenin transcriptional activity. *Right panel*, p-GSK3β levels indicate that LiCl_3_ treatment led to the phosphorylation of GSK3β. **f** Marker analysis indicates that LiCl_3_ treatment (*left panel*) induced expression of fibrotic markers whereas curcumin treatment (*right panel*) suppressed them. *Open bars*, vehicle-treated (control); *solid bars*, either LiCl_3_- or Curcumin-treated cultures. *significantly different from the control groups of the same gene at *p* < 0.05. *n* = 3. **g** Using a β-catenin inhibitor ICG-001 to block the transcriptional activity of β-catenin reduces the expression of markers for fibrosis except Timp 1. *Black bar*, DDC-treated *Pten* null mice; *gray bar*, *Pten* mull mice treated with DDC+ICG-001; *open bar*, vehicle-treated controls. *significantly different from the control groups of the same gene at *p* < 0.05. *n* = 3

To confirm the involvement of Wnt signal in HSC activation, we introduced siRNA against β-catenin, the target of Wnt to cultured rat HSCs (Fig. 9a). Inhibiting β-catenin activity led to reduced cell growth and migration (Fig. 9b, c). Particularly, significant reduction of SMAα expression (approximately threefold) was observed when β-catenin is downregulated with moderate inhibition on the expression of Col1a1 and Timp 1 (Fig. 9d). We also used chemicals that are capable of interfering with β-catenin function to modulate Wnt activities in primary HSCs and determined whether activating β-catenin activity is sufficient to promote the progression of primary HSC activation and whether β-catenin activation is necessary for this process to occur. LiCl inhibits GSK3β and blocks the degradation of Wnt downstream target β-catenin [17], whereas curcumin blocks the activity of β-catenin [18] (Fig. 9e). Consistent with a positive regulatory role of Wnt/β-catenin on fibrogenesis, LiCl_3_ treatment induced the expressions of desmin, Timp 1, and Col1a1 (Fig. 9f, left panel), whereas their expressions are moderately downregulated as a result of curcumin treatment (Fig. 9f, right panel).

Finally, we used a small molecule inhibitor of Wnt/ β-catenin, ICG-001, which specifically blocks the interaction of β-catenin with its coactivator CBP to inhibit β-catenin transcriptional activity in vivo. Fibrosis in the *Pten* null mice becomes obvious after 6 months of age. To advance this onset of fibrosis and induce the expression of Col 1a1, desmin, and Timp 1, markers for fibrosis, we used a cohort of 1.5-month-old mice with DDC (Fig. 9g). In these DDC-treated mice, ICG-001 was given to inhibit Wnt/CBP/ β-catenin transcription [19, 20]. With the exception of Timp 1, expression of all fibrogenic markers are downregulated with ICG-001 to different extent including SMAα and p75 NTR, of which the expression did no increase with DDC treatment. Expression of Col1a1, particularly, is significantly reduced whereas the change in other makers did not reach significance. While not all markers responded in a synchronized manner to LiCl, curcumin, si-β-catenin, or ICG-001, our data is consistent with a pro-fibrogenic role of Wnt in the liver. The lack of synchrony is likely due to the nature of the markers as indicators of HSC activation rather than drivers of fibrosis. Depending on the stage of activation/differentiation of HSCs, different markers are expressed, leading to the varied response to Wnt signal-induced expression changes.

## Discussion

Hepatic fibrosis is a pathological condition that follows chronic liver disease, including NASH and ASH, and is a precursor to liver cancer development. In this study, we explored the molecular link between NASH and fibrosis. We demonstrated that *Pten* null model is a relevant model for studying fibrosis development because (1) liver disease progresses similarly in humans and *Pten* null mice, (2) the *Pten* null mice develop progressive and severe fibrosis from 6 months on, and (3) HSCs implicated in fibrosis development in humans are also activated in *Pten* null mice. Enhanced expression of Col1a1, Timp 1, SMAα, and p75NTR suggests the involvement of HSCs. HSCs isolated from *Pten* null liver are fully activated with spindle morphology and low vitamin A autofluorescence, confirming the severe fibrosis phenotype that was observed in vivo.

After demonstrating the relevance of the *Pten* null mice model, we utilized these mice to investigate the signaling pathways that drive fibrosis development. We found that (1) activation of HSC is associated with steatosis and injury but not directly caused by alterations of PTEN/PI3K/AKT signaling, (2) blocking Wnt signaling significantly attenuated the ability of the quiescent HSCs to become activated, and (3) activation of Wnt signaling induces genes associated with activated HSCs. Taken together, (1), (2), and (3) suggest that Wnt activation resulting from steatosis induced liver injury activates HSCs and thus induces hepatic fibrosis.

In humans, fibrosis occurs in patients with viral infections, ASH, and NASH, as well as biliary and other diseases that lead to injury of liver parenchymals [21]. In animal experiments, fibrosis development is typically induced by inducing injury to liver parenchymals [1]. Injuries are typically induced using physical approaches such as ductal ligation and chemical approaches such as carbon tetrachloride treatment, choline deficient diet, alcoholic feedings, or ligation of the bile duct to cause acid buildup to damage the liver parenchymal [22, 23]. A similarity among these models and also a significant clinical interest is damage to hepatocytes and attenuation of their proliferation. In this study, rather than directly injuring the animal subjects using physical or chemical means, we used a NASH model where fat accumulation in the liver is a consequence of hyperactivated insulin signal, mimicking human NASH conditions [6]. Liver injury in the *Pten* null mice results from fatty liver disease much as obesity-induced fatty liver often leads to NASH in humans. We show here that NASH is accompanied by deposition of collagen fibers and activation of HSCs, and preventing NASH resolves these conditions. This study, thus, provides experimental proof that NASH does lead to fibrosis development.

AKT, the downstream kinase of PTEN, has been shown to control multiple fibrogenic genes in various tissue/cell types. In mouse skin, loss of PTEN induces the expression of fibrogenic genes [24]. Sustained expression of wild type PTEN in cultured rat HSC inhibited morphological changes associated with HSC activation [25]. Chronic activation of PI3K also induced accumulation of collagens [10, 11]. Thus, we were surprised to find that PTEN and AKT2 signals did not change significantly in HSCs isolated from the *Pten* null livers. (*Pten* deletion is liver-cell specific in *Pten* null mice. *Pten* is not deleted in HSCs.) Our observations suggest that the effect of AKT2 on fibrogenesis is likely secondary to its role in steatosis and the subsequent liver injury.

Wnt signaling has recently been reported to be important for HSC activation [13, 26]. Liver HSCs express a number of different receptors for Wnt [27]. During culturing and activation of HSCs, the expression of Wnt receptors and ligands is induced [12]. The Wnt antagonist DKK has been found to enhance the transcriptional activity of an adipogenic gene including peroxisomal proliferation activated receptor (PPARγ) [26]. Upregulation of PPARγ maintains the lipid droplets and allows the HSCs to remain quiescent with many lipid droplets present in each cell whereas Wnt blocks DKK signal, allowing activation of HSCs [14, 26]. Our study shows that this signal is likely relevant to the steatosis-induced fibrosis as several Wnt ligands and receptors are induced and sFRP5 is inhibited in the injured *Pten* null liver [6]. Whether Wnt activation is a direct consequence of steatosis or requires injury to occur remains to be determined.

## Conclusions

In summary, our data established that the liver *Pten* null model displays progressive fibrosis prior to cancer development. The fibrosis is a result of steatosis induced by PTEN loss, suggesting that the liver-specific *Pten* null mouse is a relevant model for studying the progression of liver cancer co-developed with fatty liver injury-induced fibrosis. Our data further indicates that the Wnt signal pathway likely mediates steatosis-induced fibrosis.

## Methods

### Animals

Targeted deletion of *Pten* (*Pten* null) and *Pten/Akt2* double mutant mice (Dm) were reported previously [5, 6]. Control (Con) animals are *Pten*^*loxP/loxP*^; *Alb-Cre*^*−*^. Experiments were conducted according to IACUC guidelines of the University of Southern California. Fasting glucose were measured in overnight (16 h) fasted mice. 3,5-dietoxycarbonyl-1,4 dihydrocollidine (DDC, 0.1 % *w*/*w* diet) treatment was performed in 3-month-old mice for 5 weeks. Male animals of C57BL/6 and J129svj background from the same breeding colony were used for all experiments. For 1.5-month-old mice used for ICG-001 study, DDC (0.05 % *w*/*w* diet) were given in three doses using a 2-day off 1 day on protocol. ICG-001 (5 mg/kg per day in saline) is delivered using a mini-osmotic pump that dispenses at 1 μL/h delivery rate. All experiments were conducted according to IACUC guidelines of the University of Southern California (Protocol #11162).

### Human liver samples

Human liver samples biopsied from fibrosis patients were obtained from Pennsylvania State University. All patient information was removed, and all experiments were conducted according to IRB guidelines of Pennsylvania State University.

### Database mining

Gene expression data base GSE37031 was downloaded from NCBI website. All patient information were reported in the original publication for the database [8]. The data set was then analyzed for expressions of PTEN in NASH (*n* = 8) vs. non-NASH (*n* = 7) patients. Immunohistochemical staining images were obtained from the Human Protein Atlas website (http://www.proteinatlas.org/).

### Cell lines

Rat HSCs [28] were cultured in 1 g/L glucose Dulbecco’s modified Eagle’s medium (DMEM) supplemented with 10 % fetal bovine serum (FBS), penicillin (50 units/ml), and streptomycin (50 μg/ml) at a 37 °C, 5 % CO_2_ incubator. For treatment with 0.1 μg/ml mIGF, 50 ng/ml PDGF, or 4 ng/ml TGFβ, cells were serum starved for 24 h.

### Primary hepatic stellate cell isolation

HSCs were isolated using OptiPrep gradient ultracentrifugation [12]. HSC fraction was collected from the medium and 1.034 interface of OptiPrep gradients, and cultured and morphological changes of HSCs are monitored using both light and fluorescent microscopy to monitor for vitamin A autofluorescence at indicated time points. RNAs were isolated for marker analysis to monitor the activation of HSCs. In experiment using lithium chloride (LiCl_3_), LiCl_3_ (10 mM) was added to the day 1 culture and incubated for 2 days before the collection of RNA. In experiments using curcumin, 10-μM curcumin was added to day 1 culture and RNAs were collected 2 days after the addition of curcumin. Frizzled-related protein 1 (sFRP1) (R&D systems, Minneapolis, MN) is used at 40 ng/ml added to day 3 HSC culture and morphology followed for 7 days.

### Immunohistochemistry

Liver sections were stained with hematoxylin and eosin (H&E) for morphology and Sirius Red to visualize fibrotic fibers. Sirius Red staining is quantified using Image J. Three animals per groups were quantified, and three randomly chosen areas per animal were assessed. Anti-smooth muscle actin α (SMA α) (Sigma-Aldrich) was used in indirect immunohistochemistry to further confirm the buildup of extracellular matrix.

### Quantitative PCR

Total RNA (2 μg) from liver tissues was used for qPCR analysis for gene expression. Primers used are SMA α, desmin, Col1a1, and p75NTR as previously indicated [29]. Primers for Timp 1 are: 5′-CAGTAAGGCCTGTAGCTGT GC 5′-CTCGTTGATTTCGGGGAAC. GAPDH were detected for internal controls.

### Protein electrophoresis

Protein lysates (40 μg) were loaded from each sample for electrophoresis using polyacrylamide gels. Membranes were probed with antibodies for PTEN, p-AKT, p-GSK3β, and GSK3β (cell signaling). α-Actin (Sigma) protein expression is used as loading controls.

### Statistics

Data were subjected to Student’s *t* tests for two sample comparisons. In cases of more than two groups, multivariate ANOVA was used to determine the statistical differences followed by pairwise comparison using Fischer’s LSD test. *p* ≤ 0.05 is considered to be statistically significant. Data are presented as mean ± SEM.
